# Responses to 10 common criticisms of anti-racism action in STEMM

**DOI:** 10.1371/journal.pcbi.1009141

**Published:** 2021-07-15

**Authors:** Maya L. Gosztyla, Lydia Kwong, Naomi A. Murray, Claire E. Williams, Nicholas Behnke, Porsia Curry, Kevin D. Corbett, Karen N. DSouza, Julia Gala de Pablo, Joanina Gicobi, Monica Javidnia, Navina Lotay, Sidney Madison Prescott, James P. Quinn, Zeena M. G. Rivera, Markia A. Smith, Karen T. Y. Tang, Aarya Venkat, Megan A. Yamoah

**Affiliations:** 1 Department of Cellular and Molecular Medicine, University of California San Diego, La Jolla, California, United States of America; 2 Bioethics and Science Policy Program, Duke University, Durham, North Carolina, United States of America; 3 Ecology, Evolution, and Biodiversity Program, University of California Davis, Davis, California, United States of America; 4 Department of Biology, Northeastern University, Boston, Massachusetts, United States of America; 5 Department of Food, Agricultural, and Biological Engineering, The Ohio State University, Columbus, Ohio, United States of America; 6 Porsia Curry, Black Resource Center, University of California San Diego, La Jolla, California, United States of America; 7 Mayo Clinic Graduate School of Biomedical Science, Rochester, Minnesota, United States of America; 8 School of Chemistry, University of Tokyo, Tokyo, Japan; 9 Department of Immunology, Mayo Clinic, Rochester, Minnesota, United States of America; 10 Department of Neurology, University of Rochester, Rochester, New York, United States of America; 11 Department of Chemistry, University of Toronto, Toronto, Canada; 12 Executive Women’s MBA Cohort, Women’s College, Brenau University, Gainesville, Georgia, United States of America; 13 Department of Graduate Studies, Master of Science in Legal Studies Program, Cornell Law School, Ithaca, New York, United States of America; 14 Department of Neurology, Massachusetts General Hospital, Harvard Medical School, Boston, Massachusetts, United States of America; 15 Neurosciences Interdepartmental Program, University of California Los Angeles, Los Angeles, California, United States of America; 16 Department of Pathology and Laboratory Medicine, School of Medicine, University of North Carolina at Chapel Hill, Chapel Hill, North Carolina, United States of America; 17 Department of Psychology and Neuroscience, Dalhousie University, Halifax, Canada; 18 Department of Biochemistry, University of Georgia, Athens, Georgia, United States of America; 19 Department of Physics, Massachusetts Institute of Technology, Cambridge, Massachusetts, United States of America; 20 Department of Electrical Engineering and Computer Science, Massachusetts Institute of Technology, Cambridge, Massachusetts, United States of America; Johns Hopkins University, UNITED STATES

The wrongful murders of Black individuals during 2020 (including George Floyd, Breonna Taylor, Ahmaud Aubery, and others), compounded by a long history of similar incidents, inspired protests around the world against racism and police brutality. The growing anti-racism movement sparked conversations within science, technology, engineering, mathematics, and medicine (STEMM) surrounding ways to combat racial bias in our respective fields. A spotlight was placed on the discriminatory history of scientific research and medical practice, as well as the problematic modern-day policies that perpetuate the lack of racial diversity and equity in STEMM.

While observing and participating in recent discussions about the racism that pervades institutions, departments, and scientific discourse, we have noticed a set of standard arguments against anti-racism action within STEMM. Ten of these arguments are laid out in this manuscript and paired with evidence-based counterarguments. Notably, while this manuscript is primarily centered around a United States perspective, most of our arguments and suggested actions remain applicable to other countries as well. It is crucial for a STEMM anti-racism movement to extend beyond national borders, reflecting the international nature of scientific research and collaboration.

This team of authors represents a collaboration between scientists from historically marginalized groups and their allies. By compiling published academic literature, we hope to directly confront racist ideology in STEMM with evidence-based arguments while simultaneously amplifying the research and perspectives of scholars of color. Our broad goal in articulating this information is to facilitate more productive conversations (and, in turn, tangible systemic changes) toward addressing racial discrimination within STEMM.

## 1. “There is no evidence of racism in STEMM.”

First, it is important to distinguish between the “method” of science and the “enterprise” of science [13]. The scientific method is an objective strategy to understand natural phenomena. The scientific enterprise describes how experiments are conceived, performed, and interpreted in real practice. While the scientific method is impartial, the scientific enterprise is subject to individual biases and societal values [13].

There is a large body of evidence demonstrating that racism, particularly anti-Black racism, is prevalent within the scientific enterprise. Approximately 62% of Black employees and 42% of Hispanic employees in the STEMM workforce say they have experienced racial discrimination in recruitment, the hiring process, or competing for promotions [14]. Racial discrimination often manifests as microaggressions: commonplace indignities that (whether intentional or unintentional) communicate a hostile or derogatory message to BIPOC (defined in Table 1) [5]. For example, women of color faculty report being frequently mistaken for university staff or graduate students [15], and Black professors are often perceived by their colleagues to have been hired primarily for their underrepresented minority status and feel added pressure to prove their legitimacy as a scholar [16,17].

**Table 1:** Glossary of terms.

Term	Definition
BIPOC	BIPOC stands for Black, Indigenous, and People of Color. According to the BIPOC Project, this term was created in order to “highlight the unique relationship to whiteness that Indigenous and Black people have, which shapes the experiences of and relationship to white supremacy for all people of color within a US context” [1].
Black	A person having origins in any of the Black racial groups of Africa [2].
White	A person having origins in any of the original peoples of Europe, the Middle East, or North Africa [2].
Asian	A person having origins in any of the original peoples of the Far East, Southeast Asia, or the Indian Subcontinent, including, for example, Cambodia, China, India, Japan, Korea, Malaysia, Pakistan, the Philippine Islands, Thailand, and Vietnam [2].
Hispanic	A person having origins in a Spanish-speaking country, regardless of race [2]. May or may not overlap with Latinx^1^.
Latinx or Latino/a	A person from Latin America, regardless of language or race. Latinx is the gender-neutral form of Latino/a [3]. May or may not overlap with Hispanic^1^.
American Indian or Alaska Native	A person having origins in any of the original peoples of North and South America (including Central America) who maintains cultural identification through tribal affiliation or community attachment [2].
Native Hawaiian or Other Pacific Islander	A person having origins in any of the original peoples of Hawaii, Guam, Samoa, or other Pacific Islands [2].
Individual racism	The conscious or unconscious racial prejudices held by individuals [4].
Systemic racism	The policies and practices entrenched in established institutions that result in the exclusion or promotion of designated groups [4].
Microaggression	Commonplace indignities that (whether intentional or unintentional) communicate a hostile or derogatory message to BIPOC [5].
Implicit racial bias	Unconscious prejudices against a racial group, which can influence a person’s perceptions without their awareness [6].
Racial wealth gap	The substantial, disproportionate concentration of wealth in white US families [7].
Colorism	A form of prejudice in which people with lighter skin are treated more preferentially than people with darker skin, independent of race [8].
Intersectionality	How multiple aspects of one’s identity (such as race, ethnicity, gender expression, sexual identity, class, culture, and age) contribute to compounding experiences of discrimination [9].

^1^Hispanic refers to individuals from Spanish-speaking countries, whereas Latinx (the gender-neutral form of Latino/Latina) refers to individuals from countries in Latin America. Someone can identify as both Hispanic and Latinx, or just one of them [10]. For many studies referenced in this document, Hispanic and Latinx were combined into a single category. Thus, we use “Hispanic/Latinx” when referencing the results of these studies. In cases where the study only included a Hispanic category without any mention of Latinx (or vice versa), we refer to these groups using the same language as the original study. Additionally, the US census classifies Hispanic or Latinx as an ethnicity, not a race. However, two-thirds of Hispanic/Latinx individuals in the US consider Hispanic/Latinx to be a part of their racial identity [11]. Identification as Hispanic/Latinx can also influence one’s experiences with both systemic and individual racism [12]. To account for this, we generally refer to Hispanic/Latinx as its own racial and ethnic identity, while recognizing that this categorization is imperfect and has significant overlaps with other groups. Additionally, unless otherwise specified, references to “white” people in this document do not include individuals who identify as Hispanic/Latinx.

Microaggressions are examples of individual racism, a term that describes a person’s prejudices and behaviors (both conscious and unconscious). Racism can also manifest at the systemic level. Systemic racism refers to “the policies and practices entrenched in established institutions, which result in the exclusion or promotion of designated groups” [4]. Importantly, systemic racism does not require individual intent; it is a status quo maintained by inaction and apathy, rather than overt acts of racism [4].

**Fig 1:**
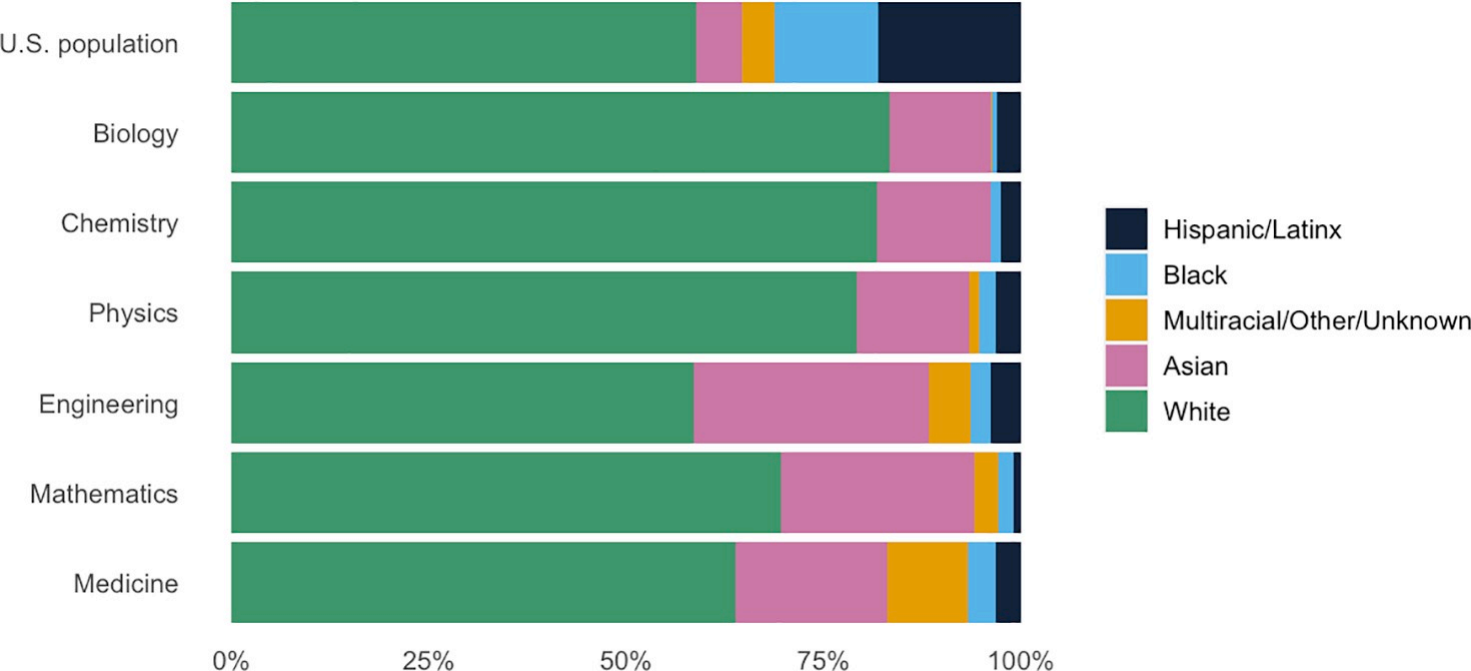
Racial/ethnic demographics of STEMM faculty. The white, Black, and Asian categories only include individuals who are not of Hispanic/Latinx ethnicity. US population data is based on estimates from the US Census Bureau as of July 1, 2019 [36]. Field-specific data are based on internal surveys recorded between 2005 and 2018 [36–40].

Black scholars are underrepresented in nearly every field of STEMM, which reflects the existence of systemic racism (Fig 1) [18]. For example, only 5.9% of noninternational students enrolled in life sciences PhD programs in the US are Black, despite making up nearly 13% of the US population [18,19]. This disparity is even greater at the college and university faculty level: Only 0.7% of biology professors are Black, while 83.3% are white [19]. Black, Latinx, and Native American professionals are also underrepresented in Big Tech companies, a trend that has improved very little in the past 5 years [20].

Systemic racism is maintained by current faculty and administration, whether consciously or unconsciously. One study found that professors rated identical CVs less favorably when the applicant had a traditionally Black or Latinx name compared to a traditionally white or Asian name [21]. Black applicants are 13% less likely than white applicants to receive research funding from the National Institutes of Health (NIH) [22]. BIPOC, especially women of color, are less likely than other groups to be invited or assigned to give talks at scientific meetings, despite requesting to present posters more often than their counterparts [23]. In the STEMM workforce, the salary levels of Black and Hispanic/Latinx professionals are substantially lower than their white counterparts at all educational levels, even when controlling for differences in field of occupation, experience, and degree-granting institution [24,25].

The existence of racism in STEMM does not only impact students and scientists. Science has a long history of mistreating Black people and other minorities as research subjects. This is evidenced by the experimental surgeries on Black enslaved women (without anesthesia) by “the father of modern gynecology” J. Marion Sims, the unconsenting and deceptive derivation and dissemination of tissue from Henrietta Lacks to create the HeLa cell line, and the blatant disregard of life and lack of consent from patients infected and untreated in the Tuskegee Syphilis Study, among others [26].

The legacy of this unethical research is still felt today, as it contributes to prevalent distrust of the medical and scientific communities by BIPOC [27]. This distrust, combined with inadequate efforts to recruit BIPOC as research subjects, means that BIPOC are underrepresented among clinical trial participants and other human research cohorts [28–30]. As a result, the benefits of our research are not distributed equally; since the large majority of research subjects and tissue donors are white (even for many diseases that disproportionately affect BIPOC), biomedical research often has reduced relevance for BIPOC [29,30]. For medical research to apply to ethnic minorities, health and research institutions must build trust and establish relationships with minorities in order to encourage participation [31].

Medical professionals today still discriminate based on false beliefs about race, which can directly impact patient care. In one study, 40% of medical students reportedly believed that Black people’s skin is thicker than white people’s. Students who held these false beliefs rated Black patients’ pain lower than white patients’ and made less accurate treatment recommendations [32]. Additionally, medical students are not taught to identify symptoms that may present differently on darker skin. When healthcare professionals are taught to identify medical conditions by the presence of rashes, skin becoming pale, or lips turning blue, BIPOC patients may be overlooked in initial screenings; their quality of care is lower even before treatment because their symptoms are less likely to be recognized [33]. Racial bias in medicine not only exacerbates distrust of biomedical research, but also entrenches systemic healthcare disparities between racial and ethnic groups [34,35].

The existence of both individual and systemic racism within STEMM institutions, as well as the role of scientists and medical professionals in perpetuating broader systems of racial inequity, have been thoroughly documented. It is our responsibility as scientists to acknowledge this body of evidence, take action to promote racial equity in our workplaces, and examine the impacts of our research on society.

## 2. “Don’t politicize STEMM! Stick to the science, not social issues.”

Scientific research is influenced by societal values and priorities. Scientists are people, which means that their opinions, biases, and beliefs will be present in STEMM workspaces—this makes science inherently political [41]. In addition, many scientific studies deal with politicized subject matter due to the implications of their findings (i.e., climate science and COVID-19) [42]. Scientific findings that have direct and immediate societal, health, or safety implications frequently require the recruitment of political institutions out of ethical concern. The goal of activism in science is to use data-backed claims to support understanding, awareness, and action in the public, political, and governmental spheres. This does not interfere with the rigor of scientific claims. If scientists avoid communicating the far-reaching implications of their science for public health and safety in an effort to solely avoid politicization, this is an ethical and professional failing [42].

Additionally, in the US and other countries, scientific research is inherently involved in politics, and public perception and support of science are divided by party affiliation [43]. This is noteworthy, as historically a quarter or greater of science funding in the US (and half of all funding for basic research) comes from government agencies [44]. The US scientific funding landscape changes with changing political party representation. In response to this, universities and scientific societies have historically lobbied in support of increased scientific funding [45]. This relationship between governmental funding, influence of science on public policy, and lobbying on behalf of scientific interests further positions science in the political realm.

Beyond institutions, scientific theories have also been used to justify racism by manufacturing biological differences between races, such as the division of human beings into racial taxa by Carl Linnaeus or the co-opting of Darwinist principles by eugenicists [46,47]. This phenomenon is not relegated to the past; even today, genetic research is often co-opted by white supremacist groups, misrepresented, and weaponized in support of racist ideas [48,49]. The biological sciences are not unique in perpetuating racism. For example, machine learning models are frequently built using racially biased datasets [50]. This has led to widely used algorithms (including for facial recognition or criminal recidivism) that are less accurate for BIPOC, perpetuating systemic racism in the criminal justice system [51–53].

Scientists are responsible for generating data and interpreting results that direct the course of many aspects of society. We have a responsibility to confront both our own biases and those present in the scientific literature in order to ensure that the data and guidance we produce are not perpetuating or enabling inequality. We must hold ourselves and our peers accountable, not only in issues of scientific integrity, but also in issues of ethical integrity.

## 3. “I’m not racist, so I don’t need to do anything.”

If you do not experience systemic racism, you are likely benefiting from it, whether by being more generously supported by your institutions, being assumed to belong, or, importantly, not bearing the significant mental and emotional burden of being subjected to racism. Racist policies only exist because those in power benefit from the oppression of the minority group. If you are benefiting from the oppression of others and choosing not to acknowledge and dismantle it, your compliance perpetuates the system. You cannot claim to be “not racist” and do nothing about it; rather, we must be actively anti-racist and take steps to combat racism in our lives (see our Call to Action) [54].

Academics who are not part of underrepresented groups can leverage their privilege to move beyond passive allyship and become active “coconspirators” against racism [55]. There are a multitude of ways to work against racism at every level of the social–ecological model; they all start with self-education on the experiences of BIPOC and self-reflection on where you can create change [56]. Self-education is crucial, as the unpaid labor for anti-racism initiatives disproportionately falls upon faculty of color, who on average spend longer than their white colleagues promoting diversity-related initiatives [57–59]. In addition, Black faculty are more likely to engage in research that addresses systemic healthcare disparities [60].

Many academics also need to bring anti-racism into their experimental design. A growing body of evidence suggests that culture and environment play a significant role in how individuals perceive themselves and their environments [61]. However, the widely held belief that human behavior, perception, and memory are innate and should not differ across cultural groups can discourage scientists from collecting or considering data about culture and ethnicity as part of their research design. This often leads to nonrepresentative studies that generalize the Western, educated, industrialized, rich, democratic experience to people from all cultures and ethnicities [61]. Researchers across all fields that study human biology and behavior have a key opportunity to exercise being inclusive and anti-racist by designing representative and robust studies.

Even if you are actively anti-racist, it is important to note that some degree of racial bias is present in all of us (even if it is subconscious), which can manifest as unintentional microaggressions rather than overt racism (discussed in Response #1) [62]. For example, faculty preconceptions of students’ learning ability—such as whether the ability to obtain and retain knowledge is fixed or flexible—can exacerbate achievement gaps in STEMM via subtle situational cues that reinforce stereotypes about which groups are more likely to excel [63]. Additionally, implicit bias can manifest at the faculty hiring level, as academic hiring committee members tend to prefer candidates who mirror their own training, values, and research interests [63]. The failure of institutions to train and continually combat implicit biases supports the continuation of practices that directly harm BIPOC. Acknowledgment of our implicit biases is necessary to continually improve teaching, mentoring, and hiring strategies at the systemic level.

## 4. “I only hire/award/cite based on merit; I do not need to consider race.”

Arguments that support the objective consideration of merit without the overarching context of external factors are often weaponized against diversity efforts. One recent example is an opinion piece by Tomas Hudlicky that was published (and quickly retracted) in the scientific journal *Angewandte Chemie* [64,65]. Hudlicky argued that the consideration of factors besides “merit” will result in candidates from underrepresented groups being chosen over more qualified candidates, thereby compromising scientific progress. (He also felt that such initiatives place nonminority candidates at an unfair disadvantage; this topic is discussed in Response #6.)

Arguments like Hudlicky’s rely on the assumption that academia is a meritocracy, wherein factors such as grades, awards, and publications depend solely on talent and effort, and therefore, we can use these metrics to objectively select the best candidate. First, it should be noted that the concept of “meritocracy” was introduced as satire in a political fiction novel by Michael Dunlop Young [66], who never intended for it to be considered as a viable sociopolitical framework [67]. Dunlop Young believed that a society structured as a meritocracy would appear equitable, but ultimately serve to reinforce and perpetuate preexisting inequality; only the most privileged would be able to access education necessary to succeed on tests of “merit,” and this could easily be weaponized to exclude those without resources [66]. As Dunlop Young understood, success in academia is highly influenced by factors outside of one’s control, including race, ethnicity, class, and gender (among many other things) [68]. Students of color are subject to both explicit and implicit acts of racial discrimination, which add an extra barrier to their success. The stress of everyday racism contributes to a heavier cognitive load for BIPOC, which is associated with both mental and physical illnesses, as well as reduced academic productivity [69,70].

**Fig 2:**
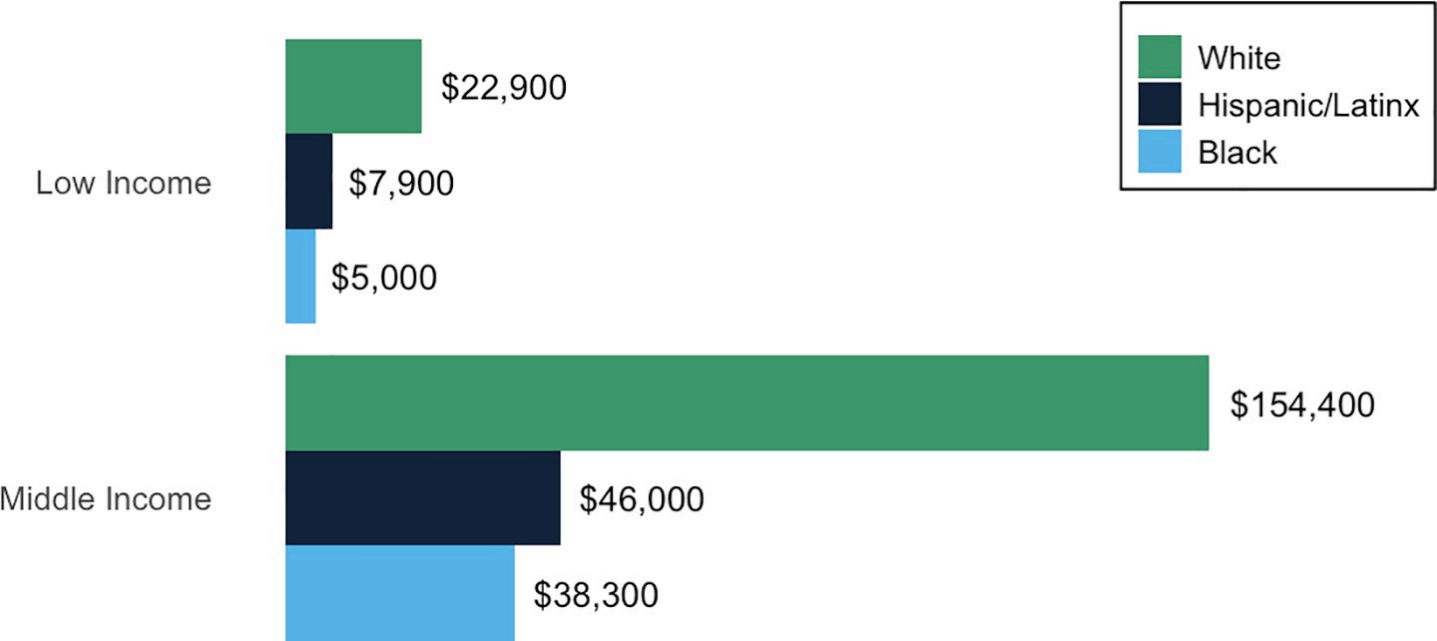
Median net worth of US households. The white and Black categories only include individuals who are not multiracial or of Hispanic/Latinx ethnicity. The high-income category contained an insufficient number of Black and Hispanic/Latinx households to be analyzed separately [7].

In addition, the wage gap (and even more so, the wealth gap) between white people and BIPOC in the US means that students of color are less likely to have financial support from their families during college [71]. The median net worth of white households is 10 times that of Black households and 8 times that of Hispanic households [7]. Even among households with the same annual income, the wealth of white households greatly exceeds that of Black and Hispanic households (Fig 2) [7]. This is because wealth takes into account the sum of a household’s total assets, including investments and real estate. Wealth accumulates over generations via gifts and inheritances. The racial wealth gap represents the legacy of slavery in the US and subsequent racist practices such as Jim Crow laws, redlining, and mass incarceration. These forces have prevented Black Americans from accumulating generational wealth while offering white Americans a 400-year head start [72].

The wealth gap contributes to unequal access to STEMM education during K-12 education (discussed in Response #7). It can also limit the ability of students of color to pursue undergraduate research, internships, and other unpaid opportunities in STEMM, or to accept low-paying positions as graduate students or postdocs. The wealth gap is also closely linked to why BIPOC tend to score lower on standardized tests, including the GRE and SAT: Individuals who identify as BIPOC may have fewer opportunities to enroll in expensive preparatory classes or to take required entrance exams multiple times [73]. In these ways, the racial wealth gap prevents a truly meritocratic system in STEMM from existing by placing disproportionate barriers on BIPOC [68].

More broadly, the criteria that define “merit” possess inherent bias. For instance, research that focuses on systemic healthcare disparities (which is more commonly pursued by Black than white researchers) is rated as less impactful by NIH grant reviewers, despite its critical importance for biomedical research [60]. Furthermore, the publications of Black scientists tend to have fewer coauthors and receive fewer citations, suggesting that Black scientists may face barriers to establishing professional networks [74]. Judging applicants on “soft skills” can also perpetuate racial bias. BIPOC at Big Tech companies are frequently perceived by their employers as not fitting in with the “workplace culture.” In many cases, BIPOC report that this perception arose from coworkers excluding them from social interactions [75].

As Malcolm Gladwell argues in *Outliers*, many highly accomplished individuals fail to recognize the degree to which privileges have contributed to their achievement, and their success is utilized as an example to justify the existence of a supposedly meritocratic system [76]. An awareness of race and its relationship to privilege reveals the infeasibility of a meritocracy. Race-conscious decision-making does not compromise our ability to select the best candidate for a position—in fact, it is quite the opposite. If our goal is to select the most talented and hard-working candidate, then we must account for the external factors that could impair an individual’s efforts from translating into standardized measures of success. Individuals who claim they are “color-blind” or “don’t see race” for the sake of upholding a nonexistent meritocracy are perpetuating the discriminatory status quo by failing to acknowledge the systemic inequities facing BIPOC [77]. We do not live in a post-racial era that is free from racial inequity and prejudice. While race itself is a social construct, the racially centered policies and organizational structures that frame our world are real. To quote Ibram X Kendi, “To be anti-racist is to focus on ending the racism that shapes the mirages, not to ignore the mirages that shape people’s lives” [54].

## 5. “There just aren’t as many BIPOC who want to work in STEMM.”

Only 14% of Black scientists and 18% of Hispanic scientists believe that a lack of interest is a major reason for the underrepresentation of Black and Hispanic individuals in STEMM [14]. However, the majority of Black and Hispanic scientists believe that a lack of educational access, discrimination in recruitment and promotions, and/or not being encouraged to pursue STEMM from an early age are the main reasons for the disparity [14]. The data back up these opinions. While it is true that a STEMM interest gap exists between white and BIPOC youth, the magnitude of this discrepancy is fairly small. A large survey of US high school seniors from 730 different schools found that 45% of white students are interested in a career related to science, compared to 40% of Hispanic students and 39% of Black students [78]. Some studies even report greater STEMM interest among BIPOC. One survey reported that Black girls in high school were more likely than white girls to say that math or computer science was their favorite subject [79].

Despite similar levels of interest, students of color face numerous barriers to achieving a career in STEMM. As discussed in Response #7, public schools that primarily serve Black students are greatly underfunded compared to primarily white schools. In addition, 39% of Black children and 35% of Hispanic children in the US are living below the poverty line (compared to 12% of white children) [80]. Black and Hispanic children are also more likely than white and Asian children to have lived through traumatic experiences [81,82]. Exposure to poverty and emotional or physical trauma during childhood are associated with an increased risk of academic, behavioral, and health problems [82]. And the effects of environmental racism can also contribute to learning difficulties [83]. For example, exposure to environmental contaminants, like lead, can affect children’s neurological development and ultimately reduce memory, cognitive function, and attention span [84,85]. Twice as many Black children have unsafe blood lead levels as non-Hispanic white children [86]. When comparing among children with unsafe blood lead levels, Black children are observed to have concentrations twice as high as white children in the same city. This effect can be detected as early as the womb [87]. Elevated lead levels are thought to be a result of exposure from household dust and paint, as well as contaminated soils and air from demolition and industrial activity. Such hazards are more commonly found in predominantly Black neighborhoods and can accumulate over generations to ultimately transmit from mothers to their children [84,87]. These factors, in combination with the racial wealth gap (discussed in Response #4), contribute to the “achievement gap”: the observation that Black and Hispanic children consistently score lower on standardized academic tests than white and Asian children [88,89].

On top of all this, the school-to-prison pipeline stymies the educational and career aspirations of many students of color. The school-to-prison pipeline is the practice by schools of removing “problem children” from classrooms as a form of punishment, which impairs their ability to keep up in school and often exacerbates behavioral problems [90,91]. Schools are also increasingly likely to involve the police in minor disciplinary incidents as part of their “zero tolerance policy.” This creates a vicious cycle that places these students on a fast track to juvenile detention, and eventually, the criminal justice system [90] The school-to-prison pipeline disproportionately affects students of color, who are more likely than white students to be suspended or expelled from school or to be arrested as children [90]. This is due to multiple factors, including racial bias among educators and school administrators, the increased risk of behavioral issues arising from childhood poverty and trauma, and schools’ emphasis on rigid obedience, which clashes with the value many BIPOC families place on questioning authority and speaking freely [92,93]. School disciplinary systems, paralleling the criminal justice system, emphasize punishment over treatment, thereby exacerbating the youth achievement gap [94].

The barriers to a STEMM career do not end once students have made it to college. Large national datasets have documented no differences between the proportions of white, Black, and Latinx students who enter college with a declared STEMM major [86]. However, Black and Latinx STEMM majors are much more likely to switch majors or drop out, demonstrating a clear issue of retention [95,96]. This pattern is not observed in the social sciences and humanities [95]. Furthermore, Black individuals are more likely than other racial groups to pursue careers unrelated to STEMM after earning their PhD in a STEMM field [97]. This “leaky pipeline” of BIPOC student disengagement is partially attributable to the scarcity of BIPOC role models among STEMM faculty; mentorship from individuals of a common racial identity has been shown to promote feelings of belonging while providing advice for navigating the unique problems that scientists of color face [98,99].

This pattern continues at the faculty level. A survey of academic search committees found that most members believed the diversity of their applicant pool was outside of their control, and made no attempt to engage applicants from diverse backgrounds [100]. Despite this widely held belief, numerous strategies have been demonstrated for enhancing the applicant pool diversity of faculty candidates, which should also be combined with more equitable hiring and retention practices [101]. Research shows that the true issue is not a lack of qualified BIPOC candidates, but a lack of active effort from academia to seek out and support these candidates.

There has been considerable research focused on the lack of supply of Black scientists as a result of the “leaky pipeline” in STEMM, but there is also a continued lack of demand for Black scientists within academia. Only 14% of Black scientists work in academia, as opposed to nearly 30% of white scientists [102]. Hypotheses to explain this disparity center around the normalized whiteness of institutions and the (often unconscious) attempts by white academics to maintain institutional whiteness and associated privilege [103]. For BIPOC, this may result in social exclusion, which would prevent access to resources and opportunities as those in power often seek newcomers who are socially similar to themselves [103]. Research suggests that this sense of social connectedness plays a key role in success, in contrast to dominant beliefs emphasizing only the role of hard work and personal motivation [104]. BIPOC are often concerned about their connectedness and perceived belonging, and feeling isolated and unsupported contributes to their increased likelihood of leaving STEMM [104].

The reasons for the underrepresentation of BIPOC in STEMM are far more complex than a lack of interest. From K-12 education up through faculty hiring, BIPOC face systemic barriers at every stage of their career, which place them at a disadvantage compared to white colleagues. Furthermore, a lack of institutional support for BIPOC results in poor retention rates at the student and faculty levels. To address these issues, we must move beyond the passive view of a “leaky pipeline” and actively dismantle the structures that limit BIPOC participation in STEMM.

## 6. “Diversity initiatives are unfair to nonminority students/faculty; it’s reverse discrimination.”

There is a widely held belief that race-conscious policies place the majority group at an unfair disadvantage. Approximately 57% of white Americans believe that white people face the same degree of racial discrimination as BIPOC, compared to only 29% of Black Americans and 38% of Hispanic Americans [105]. Belief in anti-white bias has been steadily increasing among white Americans since the civil rights movement [106]. This mindset stems from viewing racial equity and tolerance as a “zero sum game,” where more rights for BIPOC must come at the expense of white people’s rights [106]. The idea of white victimhood remains a central tenet of modern white supremacist groups [107].

Initiatives that promote racial justice and equity in STEMM are not a barrier to white scientists; rather, these programs are designed to partially remove a systemic barrier that has been placed on scientists from underrepresented groups. Ibram X. Kendi discusses this idea in *How to Be an Antiracist* [54]. Kendi argues that racial discrimination itself is not inherently racist; the question is whether the consideration of one’s race perpetuates inequity or equity. US Supreme Court Justice Harry Blackmun described it in this way: “In order to get beyond racism, we must first take account of race. There is no other way. And in order to treat some persons equally, we must treat them differently.” These initiatives are created to address long-standing barriers to entry and inequity in the STEMM and academic workplace (discussed further in Response #5). Temporarily assisting an underrepresented racial group to achieve equity is not the same thing as perpetuating inequality of wealth and power of the overrepresented group [54].

In fact, the concern that minority students are overrepresented in funding is contradicted by data showing that white students are still more likely than Black, Hispanic/Latinx, or Asian students to receive merit-based scholarships. One report found that white students receive 76% of institutional merit-based awards, despite making up only 62% of the student population [108]. Another study reported a similar trend: 16.4% of all white undergraduates are supported by at least one merit-based award, compared to only 11.6% of Black undergraduates and 8.1% of Hispanic/Latinx undergraduates [109]. Similarly, race-conscious university admissions policies have been shown to promote proportional representation of BIPOC without causing underrepresentation of white students [110]. Even the National Science Foundation’s Graduate Research Fellowship Program, which has an explicit goal of increasing diversity in STEMM, still awards 80% of its fellowships to white applicants [111].

Finally, an important part of what makes discrimination harmful is an associated power dynamic that allows the discriminator to stifle the victim. Considering that white students and faculty have maintained an exclusive community within higher education for centuries (the legacy of which still negatively impacts BIPOC), it is impossible for actions taken by and for BIPOC to amount to “reverse racism” within academia—they do not have the same powers of oppression [112–114].

## 7. “Education is the great equalizer.”

Opponents of anti-racism often assert that “education is the great equalizer.” This phrase suggests that access to education is sufficient to remove the effects of inequities between groups of people, such as barriers due to race or socioeconomic status. In other words, oppressed groups need only prioritize their education in order to pull themselves onto equal footing with more privileged demographics. Thus with the existence of free K-12 education and need-based college loans and scholarships in the US, any remaining inequities must be due to those individuals’ failure to take advantage of the available educational resources [115].

First, it’s important to recognize that access to STEMM education is not equal. For example, a large survey of US public schools found that 81% of Asian American students and 71% of white students have access to a full range of math and science courses during high school [116]. In contrast, less than half of American Indian/Native American or Native-Alaskan students have the same access, along with 57% of Black students and 67% of Latinx students [116]. This discrepancy is due in large part to “redlining,” a practice of discriminatory mortgage lending that has led to continued segregation between majority-white and majority-Black neighborhoods [117]. Since public schools are funded by local property taxes and Black households have one-tenth the median net worth of white households, school districts that primarily serve Black students are greatly underfunded [7,118,119].

The racial wealth gap (discussed in Response #4) also contributes to unequal educational access at the college level. On average, Black students take out the largest loans to complete a bachelor’s degree compared with any other racial group, putting them at a financial disadvantage after graduation [24]. Black graduates are also more likely than any other demographic to be unemployed 1 year after the completion of their bachelor’s degree [24]. This is due in part to hiring discrimination; one widely cited study found that resumes with “white-sounding” names received 50% more callbacks than identical resumes with “Black-sounding” names [120]. Another study found that companies were twice as likely to call back minority applicants who had “whitened” their resumes by concealing or downplaying any indicators of their race, as opposed to those who disclosed their race [121].

Among the employed, Black people have the lowest median annual earnings of any racial group across all education levels (from Associate’s degree to PhD), even when controlling for differences in field, experience, and degree-granting institution [24,25]. Continued disparities in income mean that education cannot act as a remedy for the wage gap experienced by Black Americans. The existence of systemic racism means that no level of education or income can truly equalize experiences and opportunities for people of color; BIPOC cannot escape pervasive discrimination by holding advanced degrees.

Rather than relying on education to be an automatic equalizer, we must examine and combat the structures that perpetuate racial inequity. In order for education to equalize, access to education, opportunities, and outcomes must first be equalized. Explicit changes must be made to address systemic racism at every level, including equitable access to K-12 education and employer-supported initiatives that increase career prospects and promote fair compensation of BIPOC in STEMM.

## 8. “I don’t agree with racist statements, but people should be allowed to express their opinions and have debates.”

The view that racist statements should be permitted as a matter not only of free speech, but also of scholarly debate, stems from a position of privilege. White people have the privilege of being able to discuss and debate racism as a detached scholarly exercise because they are not directly harmed by racism. In contrast, BIPOC have to bear the mental load of racism as a constant force in their lives; racism is not some theoretical concept, but a concrete reality, which precludes an emotionless debate. BIPOC should never be forced to explain and defend their lived experiences and racial trauma for the sake of “debate”.

Expressing racist opinions creates a hostile environment for BIPOC, which has been shown to be detrimental to their mental and physical health and success [122]. Such expressions are an example of racial microaggressions (discussed in Response #1), which reinforce themes such as “You do not belong,” “You are intellectually inferior,” and “You are abnormal” [123]. This messaging, whether covert or overt, compounds to produce an environment that can negatively impact physiological responses, self-esteem, and quality of life for BIPOC [124,125]. Perceptions of racial discrimination are related to increased rates of stress, depressive symptoms, and long-term physical health effects [126].

A truly tolerant community or workplace cannot abide by the perpetuation of racist actions or sentiments. In 1948, the United Nations General Assembly signed a Universal Declaration of Human Rights. According to Article 1 of this document and the collective agreement of the signers and their endorsing nations, the humanity and dignity of all people is simply not up for debate [127]. In 1995, the UN also released a Declaration of Principles on Tolerance, which proclaims that tolerance is not only a moral imperative, but also a political and legal requirement [128]. The declaration notes that “the practice of tolerance does not mean toleration of social injustice.” This clause upholds Karl Popper’s “paradox of tolerance”—the idea that being completely tolerant of all ideas will allow the emergence of intolerant groups, which, if left unchecked, will in turn stifle and destroy the entire framework of tolerance that permitted their formation [129]. To act in accordance with these ideas, racist sentiments cannot be tolerated; they perpetuate discrimination and injustice, which threaten a tolerant society.

In addition to its direct harm against scientists of color, humoring debates about the existence of racism can serve to legitimize racist opinions. By engaging in “scholarly” debates with racists, we acknowledge their views as worthy of attention and discussion. This carries particular weight for scientists, who are perceived as intellectual or moral authority figures by many members of the public [130,131]. Arguments that claim the nonexistence of racism are simply not worthy of debate, as their opinions are not only hateful, but also blatantly incorrect in the face of a large body of research (see Response #1).

People who deny the existence of racism are not entitled to equal time and consideration for an opinion that directly contradicts facts.

## 9. “Focusing on anti-Black racism ignores the experiences of non-Black POC, in addition to sexism, ableism, etc.”

Several recently documented instances of police brutality against Black people have sparked conversations and initiatives surrounding anti-Blackness within academia. These initiatives do not overshadow nor compete with efforts to combat sexism, ableism, or discrimination against other underrepresented minorities. Initiatives that focus on anti-Black racism simply intend to address a specific, prevalent form of racism within STEMM.

Discrimination is not monolithic, and members of different racial identities experience racism in a multitude of ways [132]. As such, addressing all forms of racism at once is not always helpful. In some cases, conflating different racial groups’ experiences of racism can cause direct harm, as in the case of the “model minority” myth that has been used to drive a wedge between the Black and Asian American communities [133,134]. Instead of viewing racism one-dimensionally, we must educate ourselves on the unique experiences of different racial and ethnic groups so that we can understand their historical and modern contexts.

One factor that influences the experience of racism is colorism, a form of prejudice in which people with lighter skin are treated more preferentially than people with darker skin [8]. Colorism exists both within and across racial groups. For example, immigrants of any racial identity with lighter skin color earn more than immigrants with darker skin [135], and the resumés of lighter-skinned Black men are preferred over darker-skinned Black men [136]. Colorism illustrates the fact that white supremacy and colonialism are built on anti-Blackness. As such, anti-Blackness serves as the basis to uphold many other forms of discrimination [137].

The concept of intersectionality is also relevant to this discussion. Intersectionality is a term coined by Kimberlé Crenshaw to describe how multiple aspects of one’s identity (such as race, ethnicity, gender expression, sexual identity, class, culture, and age) contribute to compounding experiences of discrimination [9]. In studies of intersectional inequity, racism dominates other aspects of identity in explaining instances of discrimination [138]. This does not mean that racism is the only aspect of identity that matters, nor does it mean that race is always the most important aspect of identity in any given case. Rather, it means that race remains an important lens through which to consider the compounded and intersectional experiences of BIPOC. Thus intersectionality is not only a descriptor, but serves to both explain these compounding experiences and to unite movements for anti-discrimination across group lines [138].

**Fig 3:**
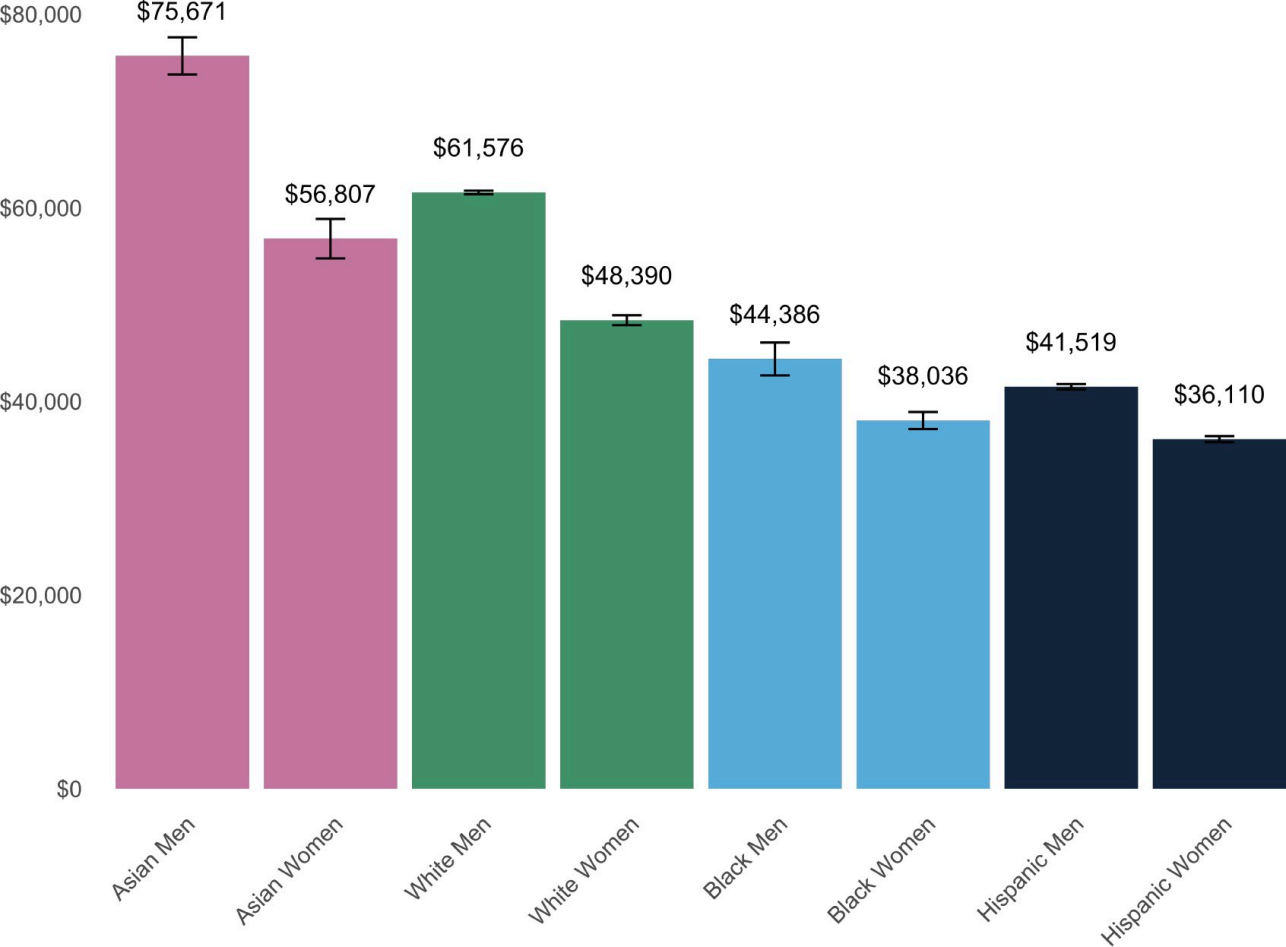
Median annual earnings by race and gender. The Asian, white, and Black categories only include individuals who are not multiracial or of Hispanic ethnicity. The Hispanic category includes individuals of any race with Hispanic ethnicity; thus, some individuals are double counted in 2 categories. Data include individuals over age 15 who were employed full-time for at least 50 weeks of the year. Error bars represent the standard error of the median [142].

By confronting anti-Blackness, validating it, and exploring its effects, we can better understand how racism and other forms of discrimination compound to produce inequality [138]. For example, any discussion of the gender wage gap is incomplete without considering intersectionality with race. White women earn 79 cents for every dollar earned by white men, but Hispanic, Native American, and Black women earn only 54, 57, or 62 cents, respectively (Fig 3) [139]. By ignoring how intersectionality contributes to experiences of discrimination, social justice movements can invalidate and exclude groups, while claiming to speak for the experiences of all in a particular group. This is evidenced in the women’s suffrage movement, which worked in opposition to the freedom of Black women [140,141]. Anti-Blackness continues to influence white feminist attitudes to this day [140,141].

To best support underrepresented scientists of all identities, it is crucial that we recognize how race drives continued marginalization and discrimination. This acknowledgment strengthens, rather than detracts from, efforts to achieve equity. In addition, to better address inequality, we must recognize how prejudice compounds through intersectionality and recruit voices from diverse backgrounds in diversity and equity efforts, so as not to paint individuals’ experiences with a broad brush.

## 10. “Improving racial equity and inclusivity does not benefit STEMM as a whole.”

Racial equity in STEMM is a moral imperative. It is the responsibility of non-BIPOC to promote distributive justice even when it does not benefit us, or when it requires us to sacrifice the unjust benefits of our racial privilege. In addition, it is important as professionals to be aware of the policies that shape our workplaces and fields and actively work to dismantle structures that are discriminatory or promote inequality. As ethical scientists, we cannot tolerate the persistence of racial discrimination in our workplaces.

Beyond being a moral obligation, promoting diversity and inclusion directly benefits all of STEMM. Diversity fosters novel ideas, effective problem-solving, and creative solutions. Given a set of problems to solve, groups that are diverse in their demographic characteristics, ethnicities, and cultural identities outperform groups that are demographically homogeneous [143]. This is true in an academic context as well; a study of more than 1 million US doctoral recipients found that students from underrepresented groups produce higher rates of scientific novelty [144]. Another study of more than 9 million scientific papers found that the ethnic diversity of the authors strongly correlated with the impact of their scientific work [145]. Scientific advancement cannot reach its full potential without uplifting and incorporating the work of people who have historically been silenced.

Furthermore, many of the complex global health issues that science aims to solve affect minority populations more severely. For example, Black individuals in the US represent a disproportionate amount of cancer diagnoses, are more susceptible to the effects of climate change, and have suffered a disproportionate amount of deaths due to COVID-19 [146–148]. As individuals whose communities are affected by these public health crises to a greater degree, BIPOC can draw on their personal experiences to offer unique perspectives on how to address them.

As a case study, one can consider climate science. Climate science and the associated environmental movement has been largely composed of white leaders [149] who have been relatively ineffective in enacting policy change [150–152]. However, a study found that Black and Hispanic people are more concerned about climate change than white people and are more likely to join campaigns in support of climate action [153]. This may be due to the different approaches between the academic climate change movement and the Environmental Justice (EJ) movement, which is founded by and primarily composed of BIPOC. The EJ movement traces its roots to Warren County, North Carolina, where protests sparked in response to a hazardous waste landfill being placed in the middle of a poor, majority Black town. Since then, EJ activists have identified and sought justice for the disproportionate burden of pollution, environmental degradation, and hazardous waste sites on communities of color [154].

In 1991, the EJ movement held a summit where hundreds of environmental leaders from indigenous communities and other communities of color joined to produce the founding documents of the EJ movement. This later resulted in the Clinton Administration’s establishment of the National Environmental Justice Advisory [155]. The EJ movement recognizes the current effects of environmental degradation and pollution, organizing efforts to address injustices that continue to disproportionately affect BIPOC, such as the Flint water crisis and the Keystone pipeline. While climate scientists have traditionally framed climate change through an academic and theoretical lens (focused on ice caps, weather patterns, and animal extinctions), the EJ movement frames climate change through a lens of personal impact, driven by grassroots organizing focused on addressing the effects of environmental racism on individuals and communities of color [149].

The EJ movement exemplifies why the inclusion of BIPOC in STEMM is so crucial. Academics and institutions, in ignoring the experiences and opinions of the people who are most affected by these issues, may continue to perpetuate perceived solutions that do not adequately address minority community needs. More concerning yet, by ignoring present issues in representation and diversity, science may produce outputs that exclude groups or perpetuate racism in society. Examples of this range from soap dispensers that are unable to recognize the presence of darker-skinned hands [156] to artificial intelligence algorithms that perpetuate racial prejudice in the criminal justice system (discussed in Response #2). A diversity of voices can help scientists to foster effective communication, design unbiased solutions, put forth just policy recommendations, and effectively recruit and engage public audiences [151].

## Conclusions and Call to Action

Both individual and systemic racism are major barriers for BIPOC in STEMM. Inequity in STEMM limits scientific innovation and upholds the broader structures of racial discrimination that exist beyond STEMM. Even in the absence of racist intent, faculty, administration, and students perpetuate racial discrimination through their inaction. It is our moral responsibility to leverage our position of privilege and become actively anti-racist.

Self-education is a necessary precursor to becoming an anti-racist, but it is only the first step towards anti-racist action. To move beyond passive “learning and listening,” we must hold ourselves, our colleagues, and our institutions accountable for promoting racial equity in concrete ways. Many BIPOC have written extensively about steps to address racism in STEMM. A few of these calls to action are outlined below and are described in greater detail in the cited resources.

- **Hire more faculty of color.** This requires actively seeking out BIPOC candidates and ensuring that the hiring process does not place BIPOC at a disadvantage, particularly through the implicit biases of the hiring committee members [100,101,157–160]. Cluster hiring, which involves advertising multiple positions simultaneously without specifying the fields, is one strategy that shows promise for improving faculty diversity [161]. In the long-term, increasing faculty diversity also requires efforts to address racial barriers that arise earlier in the educational pipeline, such as the increased likelihood for BIPOC in STEMM undergraduate majors to switch majors or drop out [95,96].
- **Create an equitable, anti-racist environment for BIPOC.** Institutions frequently prioritize recruitment over retention, which ends up doing more harm than good. It is irresponsible to recruit faculty or students of color into an unsupportive culture. Fostering an inclusive workplace culture is a complex task and must be constantly maintained to ensure retention of BIPOC. This includes providing faculty and students with diversity and inclusivity training, defending BIPOC against microaggressions, offering outlets for the negative experiences of BIPOC to be heard and addressed, hiring BIPOC mental health providers, and promptly removing individuals who create an unsafe or hostile environment for BIPOC [101,157,158,162,163].
- **Listen to BIPOC colleagues and believe them.** BIPOC who raise complaints about racial microaggressions or point out instances of structural racism are frequently disregarded for being “overly sensitive” and “making everything about race.” If BIPOC say that they have experienced racial discrimination, we must believe them and take steps to address the problem. Furthermore, individuals against whom these complaints are frequently raised should be removed from their positions of authority and not have their behavior excused with “that’s just how they are” or “they don’t mean to offend” [158,164].
- **Remove the disproportionate service burden for BIPOC.** BIPOC who take on diversity and inclusion projects should be fairly compensated for their labor, and such efforts should be recognized during decisions for hiring, promotion, and awards. Furthermore, non-BIPOC should step up to shoulder more of the workload for diversity committees and other unpaid service burdens. Note that this does not mean that white scientists should insert themselves into positions of leadership to the exclusion of BIPOC; when BIPOC organizers are present, allies should listen to their ideas and volunteer to contribute where help is needed. Non-BIPOC who participate in diversity initiatives should prioritize listening to BIPOC colleagues and trainees to ensure that their needs are being heard and solutions are oriented towards their benefit [58,158,165].
- **Allocate funding at the institutional level toward diversity initiatives.** These may include scholarships and travel grants for students of color, BIPOC mentorship and professional development programs, seminars highlighting the research of BIPOC, and diversity training courses for faculty and students. Hire experts in diversity, equity, and inclusion to facilitate this work, rather than expecting BIPOC to provide unpaid labor [158,166,167].
- **Reject “color-blindness” and maintain an awareness of racial inequities in all contexts.** Notice when BIPOC are underrepresented among grant or scholarship awardees, positions of leadership, conference panelists, or research study cohorts. Do not leave it to BIPOC to bring attention to these issues; point out instances of inequity and seek to address them directly [158,164,167,168].
- **Incorporate anti-racist principles into your pedagogy.** Highlight the contributions of BIPOC to your field and point out instances where scientific research has been used to perpetuate racial discrimination. Educate the next generation of STEMM leaders to be aware of racial inequity and empower them to take action [158,169,170].
- **Accept criticism with grace and use it to grow.** If a BIPOC colleague or student takes offense to your words or actions, do not try to defend yourself or justify your actions. Instead, apologize and use this as an opportunity to examine your own biases and improve in the future. Remember, your intent does not matter; it is the impact of your actions that matters [158,171,172].
